# Cross-sectional study of the association between diet and physical inactivity with obesity, diabetes and hypertension among older adults in Sierra Leone

**DOI:** 10.1136/bmjopen-2024-095825

**Published:** 2025-07-01

**Authors:** Tahir Bockarie, Ankit Shanker, Mohamed B Jalloh, Alhaji M Kamara, Maria Lisa Odland, Haja Wurie, Rashid Ansumana, Joseph Lamin, Miles Witham, Oyinlola Oyebode, Justine Davies

**Affiliations:** 1 Faculty of Life Sciences & Medicine, King's College London, London, UK; 2 Warwick Medical School, University of Warwick, Coventry, UK; 3 University of Sierra Leone College of Medicine and Allied Health Sciences, Freetown, Sierra Leone; 4 Nuffield Department of Medicine, University of Oxford, Oxford, UK; 5 Heidelberg University, Heidelberg, Germany; 6 Institute of Applied Health Research, University of Birmingham, Birmingham, UK; 7 University of Sierra Leone, Freetown, Sierra Leone; 8 Bo Campus, Bo, Sierra Leone; 9 Mercy Hospital Research Laboratory, Freetown, Sierra Leone; 10 NIHR Newcastle Biomedical Research Centre, Newcastle University, Newcastle upon Tyne, UK; 11 Queen Mary University of London, London, UK; 12 Global Health, University of Birmingham, Birmingham, UK

**Keywords:** Cardiovascular Disease, Risk Factors, Physical Fitness, Obesity

## Abstract

**Abstract:**

**Objective:**

To examine the association between behavioural risk factors and their physiological sequelae among adults aged 40 and above in Bo District, Sierra Leone.

**Design:**

Cross-sectional study.

**Setting:**

Household survey in Bo District, Sierra Leone.

**Participants:**

The study included 1978 randomly sampled adults aged 40 and above (44.4% male and 55.6% female). The majority of participants were aged 40–49 years (34.5%). Data were collected using a household survey based on the validated WHO STEPs questionnaire.

**Methods:**

Multivariable logistic regression analysis was performed to determine associations between behavioural risk factors (diet, physical activity and salt intake) and the presence of hypertension, diabetes and/or obesity, adjusting for sociodemographic variables.

**Primary outcome measure:**

The primary outcomes were the presence of hypertension, diabetes or overweight/obesity. Hypertension was defined as systolic blood pressure of ≥140 mm Hg and/or diastolic blood pressure of ≥90 (measured); diabetes as fasting glucose of ≥7.0 mmol/L, random plasma glucose level of ≥11.1 mmol/L or the use of antidiabetic medications (self-reported) and overweight/obesity as having a body mass index of ≥25 kg/m² (measured).

**Results:**

At least one physiological risk factor for cardiovascular diseases, that is, hypertension, obesity or diabetes, was present in 43.5% of participants. Hypertension was associated with urban living (OR=1.46, 95% CI (1.41 to 1.51)), older age (OR for 80+=3.98, 95% CI (3.70 to 4.28)), insufficient fruit and vegetable intake (OR=1.52, 95% CI (1.46 to 1.60)) and low physical activity (OR=1.35, 95% CI (1.27 to 1.43)). Diabetes was associated with urban residence (OR=1.84, 95% CI (1.66 to 2.05)), older age (OR for 70–79=3.82, 95% CI (3.28 to 4.45)), low fruit and vegetable consumption (OR=1.61, 95% CI (1.36 to 1.90)), high salt intake (OR=1.34, 95% CI (1.21 to 1.49)) and low physical activity (OR=1.47, 95% CI (1.26 to 1.71)). Obesity was associated with urban living (OR=1.66, 95% CI (1.59 to 1.72)), high salt intake from two or more sources (OR=1.21, 95% CI (1.17 to 1.25)) and low physical activity (OR=1.30, 95% CI (1.22 to 1.39)). Male sex (OR=0.37, 95% CI (0.36 to 0.38)) and older age (OR for 80+=0.39, 95% CI (0.35 to 0.43)) were protective factors.

**Conclusions:**

In Bo District, nearly half of adults over 40 face hypertension, diabetes or obesity, especially urban dwellers, older age groups and those eating too few fruits and vegetables, consuming excess salt and getting little exercise. Public health efforts should focus on urban‐targeted nutrition education, salt‐reduction strategies, community exercise programmes and routine blood pressure and glucose screening, working with local leaders to ensure sustainable lifestyle changes and early disease detection.

**STRENGTHS AND LIMITATIONS OF THIS STUDY**

- This study used a validated WHO STEPs questionnaire to ensure standardised data collection on behavioural and physiological risk factors.
- The sampling strategy included both rural and urban communities, enhancing population diversity.
- A cross-sectional design was used, which limits the ability to infer causal relationships.
- Reliance on self-reported measures may introduce reporting bias and measurement inaccuracies.
- The study’s geographic focus on Bo District may limit the generalisability of findings to other regions in Sierra Leone.

## Introduction

 Non-communicable diseases (NCDs), including cardiovascular disease (CVD), are the leading causes of mortality worldwide, accounting for 78% of global deaths, constituting a significant public health challenge.^1^,^4^ In 2019, CVDs alone were responsible for 17.9 million deaths globally, underscoring their significant public health relevance.^5 6^ Low-income and middle-income countries (LMICs) face a dual burden of infectious and NCDs, straining healthcare systems and hindering economic development.^7^ Research has highlighted that both LMICs and the vulnerable communities of high-income countries (HICs) share a disproportionate burden of CVDs.^6^ This threatens progress towards Sustainable Development Goal 3 (SDG3)—‘Ensure healthy lives and promote well-being for all at all ages by 2030’, 1 of the 17 SDGs established by the United Nations in 2015.^3 7^ Behavioural risk factors—unhealthy diets, physical inactivity, tobacco use and harmful alcohol consumption—are the primary drivers of physiological conditions such as hypertension, diabetes and obesity.^48^,^10^ Understanding the interplay between these behavioural and physiological risk factors in different populations is crucial for developing effective public health strategies aimed at mitigating the prevalence of CVDs. Systematic reviews have highlighted that salt intake in sub-Saharan Africa (SSA) is alarmingly high, often exceeding recommended levels due to the consumption of processed foods and added salt. Unhealthy diets, characterised by high consumption of processed foods, sugars, and salt, and low intake of fruits and vegetables, are closely linked to the development of hypertension and obesity.^11 12^

Although there is growing understanding of behavioural risk factors for CVDs in many LMICs, there is less knowledge about these factors in some of the most deprived countries in SSA. This gap is concerning, especially given the rising prevalence of CVDs in these regions amid rapid demographic shifts. Urbanisation often leads to lifestyle changes including increased consumption of processed foods and reduced physical activity due to greater reliance on motorised transportation.^13^,^17^ Studies indicate that urban dwellers in LMICs exhibit dietary patterns characterised by insufficient intake of fruits and vegetables, high consumption of sugar and salt, and increased intake of vegetable oil, meat and processed foods.^1011 18^,^22^ These eating patterns—too much sugar, salt, vegetable oil, meat and other processed foods, and too few fruits and vegetables—form an unhealthy diet that is driving the rising rates of hypertension and obesity in LMIC.^10 18^ Furthermore, research on dietary consumption patterns in SSA reveals a complex interaction between meat, fruit and vegetable intake. While meat consumption has increased, often driven by urbanisation and higher income levels, fruit and vegetable intake remains critically low. This imbalance in dietary patterns is linked to rising NCDs and underscores the need for targeted nutritional interventions.^12^ Earlier systematic reviews confirm that excessive dietary salt, low fruit-and-vegetable intake and physical inactivity are major behavioural drivers of CVD in LMICs, yet they also reveal how little integrated, country-specific evidence exists: fewer than 15 cross-sectional surveys have linked plant-based diets to any physiological marker beyond body mass index (BMI), and just a handful of nations have collected the full suite of diet, activity and clinical measurements in the same population.^23^,^26^ To address this gap, we conducted a WHO-STEPS household survey in Bo District, Sierra Leone, obtaining linked behavioural and physiological data from 1978 adults aged ≥40 years. We quantified the independent and combined effects of inadequate fruit-and-vegetable consumption, high discretionary salt use and insufficient physical activity on objectively measured hypertension, diabetes and obesity, providing the contemporary, locally grounded estimates that national policymakers need for setting nutrient targets, designing food-environment regulations and prioritising activity-promotion interventions.

## Methods

### Study setting

This study was conducted in Bo District, situated in the Southern Province of Sierra Leone. Bo District includes both well-defined rural areas and Bo City, the country’s second-largest urban centre. Unlike the Western Area, which is predominantly urban, Bo District presents a balance between rural and urban settings, making it a representative location for studying population health trends. According to the 2015 census, Bo District had a population of 575 478, with 380 307 (66.1%) residing in rural areas and 195 171 (33.9%) living in urban areas. Among the total population, 17.4% (100 188) were over 40 years or older.^27^

### Sampling strategy

A cross-sectional household survey was conducted from September to December 2018 to assess CVD risk factors, with particular attention to diabetes prevalence, which was expected to be the least common of the three key outcomes (hypertension, diabetes and obesity). This informed the sample size calculation, as detecting diabetes with adequate statistical precision required the largest sample. The initial target sample size was 1893 participants, calculated to detect a diabetes prevalence of 4% with ±1% precision. To account for potential non-response and missing data, an oversampling rate of 10% was applied, bringing the recruitment target to approximately 2082 individuals.

**Figure 1:**
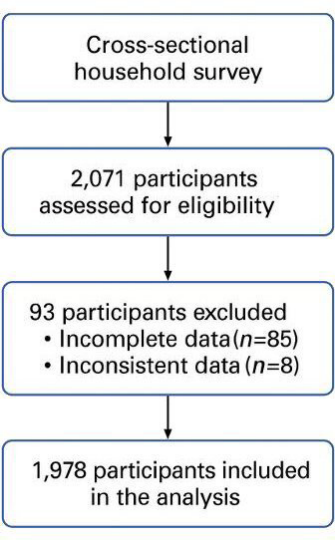
Participant flow diagram.

During data collection, a total of 2071 participants aged 40 years and older were successfully recruited across both urban and rural communities. Following data cleaning, 93 participants were excluded due to incomplete or inconsistent data, resulting in a final analytical sample of 1978 individuals (see figure 1).

Participants in this study were individuals aged 40 years and older, selected from both rural and urban areas. The sampling proportions were aligned with the habitation patterns documented in the 2015 population and housing census for Bo District. This alignment ensured that the sample was representative of the population distribution in terms of age and urban–rural residency. Exclusion criteria included confirmed pregnancy and diagnosis with a terminal or incapacitating illness, which could have potentially influenced the study’s outcomes and participant availability.

To reflect the urban–rural population ratio, the study aimed to include 700 urban and 1300 rural participants. We purposively sampled the only two urban chiefdoms (Bo City and Tikonko). Out of the 24 eligible urban communities within Bo City and Tikonko, 7 were selected from a randomly ordered list, with 100 participants recruited in each. For the rural sample, 7 out of the 14 rural chiefdoms were randomly selected using a random ordering method. We aimed to recruit 93 participants from each selected settlement or village. If the target number was not achieved in the initial community, additional communities were drawn from a prerandomised list until the quota was reached.

In each community, data collection followed a systematic approach, ensuring comprehensive coverage and accuracy. Data collectors began at randomly chosen points within each community and sampled every second household along roads or tracks. Each household was permitted to enrol a maximum of two eligible individuals aged 40 years and older. In smaller communities with fewer than 93 eligible individuals, all individuals over 40 years were included in the study. If a selected participant declined to participate, another individual within the household or community was chosen. In cases where no one was home, a message was left with neighbours, and the researchers returned the following day to complete the sampling.

The study’s geographical scope was limited to a 40 km radius from the centre of Bo, ensuring accessibility for researchers and efficient data collection. All chiefdoms and subdistricts in Bo District fell within this radius, allowing for a comprehensive and representative sample of the district’s population.

### Data collection

Data were collected electronically using ODK (Open Data Kit) software on tablet devices by a team of 15 trained data collectors. The survey was based on the WHO’s STEPwise approach to chronic disease risk factor surveillance (WHO-STEPS). WHO-STEPS methodology provides a standardised framework for collecting, analysing and disseminating data on NCDs and their associated risk factors.

The household survey incorporated all three steps of the WHO-STEPS approach:

1. Step 1: Questionnaire—A comprehensive questionnaire was administered, covering sociodemographic characteristics, behavioural and dietary habits, and medical history related to hypertension and diabetes. The demographic section included questions on age, sex, marital status and education, while behavioural queries addressed fruit and vegetable intake, physical activity levels, salt intake and smoking habits. The medical history component inquired about past incidences of stroke, heart attack or angina, as well as previous treatments for raised blood pressure and cholesterol levels. Additionally, the questionnaire included 49 items related to household assets, construction materials and awareness of CVD risk factors and treatments.
2. Step 2: Physical measurements—Physical measurements were conducted to obtain anthropometric data and blood pressure readings. Height was measured with a measuring stick with participants standing without shoes, backs, hips, and heels against a wall, and looking ahead horizontally. This method was validated using the SECA 213 stadiometer during training and biweekly checks. Body weight was recorded using an Omron medical scale, calibrated daily. BMI was calculated as weight in kilograms divided by the square of the height in metres (kg/m²). Blood pressure was measured using an Omron M6 AC LED blood pressure monitor, with three readings taken 5 min apart; the average of the last two readings was recorded. Participants were seated and rested during these measurements.
3. Step 3: Biochemical measurements—Blood samples were obtained in the morning after an 8-hour overnight fast. The Accutrend Plus point-of-care device (Roche Diagnostics) was used to measure fasting capillary glucose concentrations; results were converted to plasma glucose levels using a conversion factor of 1.11. Participants’ fasting status was verified prior to blood sampling, and those who had not fasted were labelled accordingly.

### Demographic information

Demographic information included geographical area, sex and age. Geographical area was categorised as either rural or urban, sex was classified as female or male. Age was recorded both as a continuous variable and presented as a categorical variable, divided into five groups: 40–49 years, 50–59 years, 60–69 years, 70–79 years and 80+years.

### Outcome measures

The study focused on three primary outcome measures: overweight or obesity, hypertension and diabetes. Overweight or obesity was defined as having a BMI of ≥25 kg/m², while a BMI <25 kg/m² was used as the reference category. Hypertension was defined by a systolic blood pressure of ≥140 mm Hg or a diastolic blood pressure of ≥90 mm Hg, or current use of blood pressure-lowering medication. Diabetes was identified by a fasting plasma glucose level of ≥7.0 mmol/L, a random plasma glucose level of ≥11.1 mmol/L or the use of antidiabetic medications.

### Behavioural risk factors

Behavioural risk factors included dietary habits, specifically fruit, vegetable and salt intake, and physical activity levels. Consumption of fruits and vegetables was reported as servings using show cards provided by data collectors. Low consumption was defined as fewer than five servings daily (<5 FV), aligning with global dietary recommendations for fruit and vegetable intake. For salt intake, participants could select from four responses: (1) added salt at the table, (2) added salt during cooking, (3) consumed salty snacks or (4) did not use salt from any of these sources. Responses were categorised into three groups: those who consumed salt from only one source (either table salt, cooking salt or salty snacks), those who consumed salt from two or more sources and those who did not use salt from any of the specified sources.

Physical activity levels were assessed based on self-reported hours of activity per week, in accordance with previous studies.^22^ Total hours and minutes of activity across work, travel and leisure domains were aggregated into two categories: less than 150 min per week (inadequate moderate and vigorous physical activity (MVPA)) and 150 min or more per week (adequate MVPA). Physical inactivity was assessed by asking participants, “Excluding sleeping, how much time do you usually spend sitting or reclining on a typical day?” The total daily hours were then categorised into two groups: less than 3 hours and 3 or more hours. This cut-off was chosen based on prior studies that have identified sedentary behaviour exceeding 3 hours per day as being associated with increased cardiometabolic risk.^28 29^ A composite variable was created to capture overall physical activity risk. Participants were categorised into three groups: no risk factors (adequate MVPA and less than 3 hours of daily inactivity), one risk factor (either inadequate MVPA or 3 or more hours of daily inactivity) and two risk factors (both inadequate MVPA and 3 or more hours of daily inactivity).

To evaluate the combined impact of multiple behavioural risk factors, we created a compound variable ranging from 0 to 6, reflecting the number of risk factors each individual exhibited, with risk factors aligned to the definitions above. The behavioural risk factors considered were: (1) insufficient fruit and vegetable consumption (0 or 1); (2) salt intake from (0 (no sources), 1 (just one source), 2 (for consumption of salt from two or three sources, where the sources are table salt, cooking salt and salty snacks)), (3) low physical activity (0 for adequate MVPA and less than 3 hours of daily inactivity, 1 for either inadequate MVPA or 3 or more hours of daily inactivity and 2 for having both physical activity risk factors (both inadequate MVPA and 3 or more hours of daily inactivity). Participants were assigned scores based on the cumulative presence of these risk factors. A score of 0 was assigned to individuals with no risk factors, while a score of 6 was assigned to those exhibiting all six risk factors.

### Patient and public involvement

Patients and/or the public were not involved in the design, conduct, reporting or dissemination plans of this research.

### Statistical analysis

Statistical analyses were performed using the SPSS V.27 (IBM). Participants with missing data on lifestyle factors, outcome measures or covariates were excluded from the analysis. This accounted for 93 individuals (approximately 4.5% of the total recruited sample), resulting in a final analytic sample of 1978 participants. Descriptive statistics, including frequencies (proportions) and means with SD, were used to summarise the data. The data were weighted using probability weights based on age and sex distributions of the adult population (aged 40 and above) from the 2015 Population and Housing Census for Bo District, to ensure representativeness. The total weighted population reflected in the analysis was 96 956 individuals. A p≤0.05 was considered statistically significant.

Multivariable adjusted binary logistic regression models were used to analyse the associations between behavioural risk factors and CVD risk factors (hypertension, diabetes and obesity). The models incorporated all behavioural and sociodemographic covariates simultaneously, without using stepwise selection. This approach ensured a comprehensive evaluation of the relationships between behaviours and health outcomes.

## Results

### Summary of population characteristics, cardiovascular conditions and risk factors

After excluding 93 cases due to missing values, the final sample consisted of 1978 individuals. The sociodemographic characteristics of the unweighted and weighted study populations are presented in table 1.

**Table 1:** Sociodemographic characteristics of study population

Household survey participants		
	Unweighted	Weighted
	n (%)	n (%)
Location		
Rural	1205 (60.9)	60 739 (62.6)
Urban	773 (39.1)	36 217 (37.4)
Sex		
Female	1100 (55.6)	47 766 (49.3)
Male	878 (44.4)	49 190 (50.7)
Age group		
40–49	682 (34.5)	43 116 (44.5)
50–59	546 (27.6)	24 379 (25.1)
60–69	340 (17.2)	14 381 (14.8)
70–79	251 (12.7)	8913 (9.2)
80+	159 (8.0)	6166 (6.4)
Education		
Some education	615 (31.1)	31 731 (32.7)
No education	1363 (68.9)	65 225 (67.3)
Marital status		
Married or cohabiting	1355 (68.5)	70 364 (72.6)
Not married	623 (31.5)	26 591 (27.4)
Wealth quintile		
Poorest	395 (20.0)	19 896 (20.5)
Poorer	397 (20.1)	19 964 (20.6)
Middle	397 (20.1)	19 493 (20.1)
Richer	394 (19.9)	19 093 (19.7)
Richest	395 (20.0)	18 510 (19.1)

Among the unweighted study participants, there were 878 men (44.4%) and 1100 women (55.6%). The majority of participants were between 40 and 49 years old (34.5%), had no formal education (68.9%) and were married or cohabiting (68.5%). The weighted study population characteristics were similar to those of the unweighted population. For a descriptive analysis of behavioural risk factors and CVD physiological risk factors, see online supplemental table 1 and for a descriptive analysis of sociodemographic characteristics by hypertension, diabetes and weight status, see online supplemental table 2.

**Figure 2:**
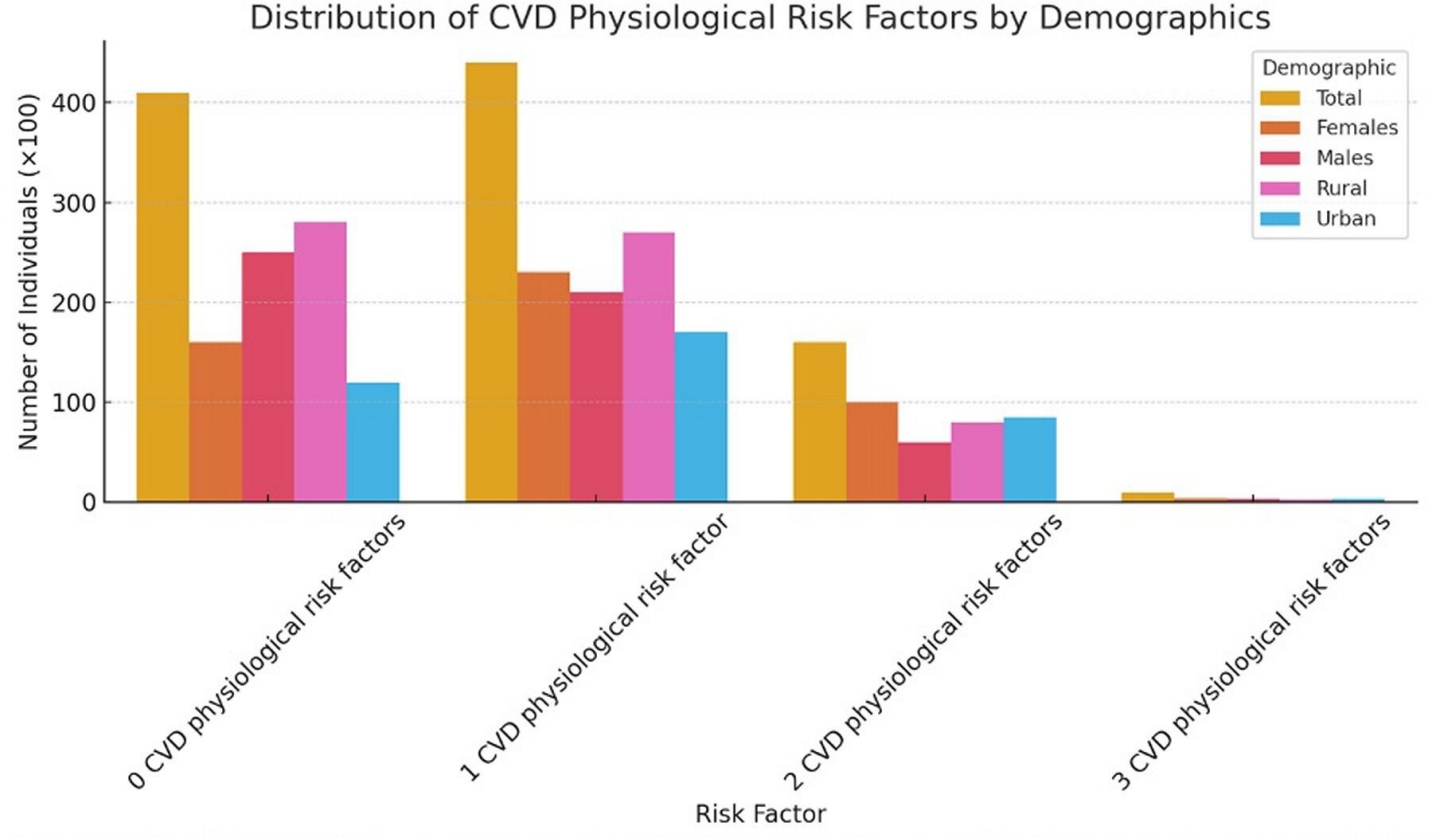
CVD physiological risk factors by sex and location. CVD, cardiovascular disease.

Out of the 1978 participants, 43.5% had at least one physiological risk factor for CVD, as shown in table 2. A higher proportion of females (45.9%) compared with males (41.3%) reported having at least one of these conditions (see figure 2). Additionally, fewer rural dwellers (42.6%) had at least one condition compared with urban dwellers (45.0%).

**Table 2:** Summary of number of co-occurring physiological risk factors by sex and by location

Multiple physiological risk factors	TotalN (%)	FemalesN (%)	MalesN (%)	RuralN (%)	UrbanN (%)
0 CVD physiological risk factors	40 820 (40.3)	15 960 (32.2)	24 860 (48.2)	28 963 (45.5)	11 858 (31.6)
1 CVD physiological risk factor	44 060 (43.5)	22 764 (45.9)	21 296 (41.3)	27 171 (42.6)	16 889 (45.0)
2 CVD physiological risk factors	15 598 (15.4)	10 310 (20.8)	5288 (10.2)	7377 (11.6)	8221 (21.9)
3 CVD physiological risk factors	747 (0.7)	588 (1.2)	159 (0.3)	198 (0.3)	549 (1.5)

CVD, cardiovascular disease.

Table 3 shows an increase in the odds of CVD physiological risk factors (hypertension and obesity) with an increasing number of behavioural factors. Individuals with six behavioural risk factors had a significantly greater risk of hypertension (OR=2.15, 95% CI (1.88 to 2.44), p<0.001) and overweight/obesity (OR=2.41, 95% CI (2.09 to 2.78), p<0.001) compared with those in the reference category. For hypertension and obesity, a clear dose-dependent association was observed. Diabetes was not included in the table as the association with increasing behavioural risk factors was not statistically significant. Including non-significant results was deemed unlikely to enhance interpretation and was therefore omitted for clarity and focus.

**Table 3:** Association between multiple behavioural risk factors and physiological risk factors

Number of risk factors	Hypertension*OR (95% CI)	Obesity*OR (95% CI)
0–1 (reference category)	Referent	Referent
2	1.01 (0.96 to 1.05)	1.61*** (1.52 to 1.70)
3	1.17*** (1.12 to 1.22)	1.70*** (1.61 to 1.80)
4	1.40*** (1.33 to 1.47)	2.05*** (1.93 to 2.17)
5	1.81*** (1.69 to 1.94)	2.40*** (2.21 to 2.60)
6	2.15*** (1.88 to 2.44)	2.41*** (2.09 to 2.78)

* Significance levels are denoted by stars: ***p≤0.01, **0.01<p≤0.05 and *0.05<p≤0.10.

Risk factors include insufficient fruit and vegetable consumption (<5 servings/day), inadequate salt intake (from one source), high salt intake (from 2 to 3 sources), low physical activity (<3 hours/day), one physical activity risk factor and two physical activity risk factors.

Diabetes was not shown due to lack of statistically significant association across behavioural risk factor categories.

In the multivariable logistic regression models individuals living in urban areas (OR=1.46, 95% CI (1.41 to 1.51), p<0.001), increasing age (OR for 80+=3.98, 95% CI (3.70 to 4.28), p<0.001), consuming fewer than five servings of fruits and vegetables daily (OR=1.52, 95% CI (1.46 to 1.60), p<0.001) and having two physical activity risk factors (OR=1.35, 95% CI (1.27 to 1.43), p<0.001) had significantly higher odds of hypertension (see table 4). For the unadjusted logistic regression models for behavioural risk factors and physiological conditions, see online supplemental table 3.

**Table 4:** Adjusted logistic regression model between multiple behavioural risk factors and CVD physiological risk factors

	Hypertension*OR (95% CI)	Diabetes*OR (95% CI)	Overweight/obesity*OR (95% CI)
Location			
Rural	Referent	Referent	Referent
Urban	1.46*** (1.41 to 1.51)	1.84*** (1.66 to 2.05)	1.66*** (1.59 to 1.72)
Sex			
Female	Referent	Referent	Referent
Male	0.84*** (0.81 to 0.87)	0.90 (0.81 to 1.01)	0.37*** (0.36 to 0.38)
Age group			
40–49	Referent	Referent	Referent
50–59	2.20*** (2.02 to 2.18)	0.98*** (0.83 to 1.16)	1.01 (0.97 to 1.06)
60–69	3.02*** (2.88 to 3.17)	3.42*** (2.98 to 3.93)	0.99 (0.94 to 1.04)
70–79	3.74*** (3.53 to 3.97)	3.82*** (3.28 to 4.45)	0.55*** (0.51 to 0.59)
80+	3.98*** (3.70 to 4.28)	1.64*** (1.31 to 2.05)	0.39 *** (0.35 to 0.43)
Fruit and vegetables			
>5FVeg	Referent	Referent	Referent
<5FVeg	1.52*** (1.46 to 1.60)	1.61*** (1.36 to 1.90)	0.99 (0.94 to 1.04)
Salt intake			
<1 source of salt intake	Referent	Referent	Referent
2 or 3 sources of salt intake	0.94*** (0.91 to 0.97)	1.34*** (1.21 to 1.49)	1.21*** (1.17 to 1.25)
Physical activity risk factors			
0 Physical activity risk factors	Referent	Referent	Referent
1 Physical activity risk factors	1.22*** (1.18 to 1.26)	0.94 (0.84 to 1.06)	1.12*** (1.08 to 1.16)
2 Physical activity risk factors	1.35*** (1.27 to 1.43)	1.47*** (1.26 to 1.71)	1.30*** (1.22 to 1.39)

* Significance levels are denoted by stars: ***p≤0.01, **0.01<p≤0.05 and *0.05<p≤0.10.

CVD, cardiovascular disease.

Higher odds of diabetes were observed among individuals living in urban areas (OR=1.84, 95% CI (1.66 to 2.05), p<0.001), increasing age (OR for 70–79 years=3.82, 95% CI (3.28 to 4.45), p<0.001), consuming fewer than five servings of fruits and vegetables daily (OR=1.61, 95% CI (1.36 to 1.90), p<0.001), consuming salt from two or three sources (OR=1.34, 95% CI (1.21 to 1.49), p<0.001) and having two physical activity risk factors (OR=1.47, 95% CI (1.26 to 1.71), p<0.001).

Urban residents showed higher odds of overweight/obesity (OR=1.66, 95% CI (1.59 to 1.72), p<0.001), as did those consuming salt from two or three sources (OR=1.21, 95% CI (1.17 to 1.25), p<0.001) and those with two physical activity risk factors (OR=1.30, 95% CI (1.22 to 1.39), p<0.001). Conversely, male individuals (OR=0.37, 95% CI (0.36 to 0.38), p<0.001) and older age groups (70–79 years: OR=0.55, 95% CI (0.51 to 0.59), p<0.001; 80+ years: OR=0.39, 95% CI (0.35 to 0.43), p<0.001) had significantly lower odds of overweight/obesity.

## Discussion

This study identified significant associations between various behavioural and physiological risk factors for hypertension, diabetes and obesity, highlighting important public health implications for Sierra Leone. These findings have important public health implications for Sierra Leone, where CVD and its risk factors are becoming increasingly prevalent.^30^ The results underscore the necessity of reducing poor dietary habits and increasing physical activity to mitigate the onset of these chronic conditions, aligning with global and regional health priorities such as Goal 3 of SDGs 2030. Furthermore, these findings align with the urgent need for enhanced postdischarge care and reinforced secondary prevention strategies in Sierra Leone, given the dire long-term outcomes for stroke survivors in the country.^31^

Consistent with existing literature, our results underscore the protective role of adequate fruit and vegetable intake. Participants who consumed fewer than five servings of fruits and vegetables daily were found to have a higher likelihood of experiencing hypertension, diabetes and overweight/obesity. This pattern highlights the significant health risks associated with inadequate fruit and vegetable intake. This finding aligns with prior studies that have demonstrated the association between higher fruit and vegetable consumption and reduced risk of CVD, likely due to their high content of dietary fibre, vitamins and minerals such as potassium and magnesium.^32^,^36^

Unexpectedly, our study found that higher salt intake was associated with lower odds of hypertension but higher odds of diabetes and overweight/obesity. This finding contradicts established research.^37^ Several factors could explain this surprising result. First, the cross-sectional nature of our study limits our ability to determine causality. It is possible that individuals who were already aware of their hypertension were actively reducing their salt intake at the time of the survey, leading to a reverse causation effect. Second, dietary assessment relies on self-reporting, which is subject to biases. It is also possible that higher salt intake is associated with other dietary habits that promote diabetes and obesity, such as increased consumption of processed foods. Salt intake has been linked to increased risk of hypercholesterolaemia in people with diabetes.^38^ Further research is needed to explore this relationship in the Sierra Leonean context, including longitudinal studies and more objective measures of sodium intake. Salt sensitivity is common among people of African descent, which makes it unlikely that this finding is a causal relationship.^39^,^41^ The association between physical inactivity and increased risk of diabetes and obesity is well-documented.^42^,^47^ Our study supports these findings, showing that participants with two physical activity risk factors had higher odds of diabetes and overweight/obesity. This is consistent with studies conducted in West African populations and underscores the need for interventions promoting physical activity to reduce the prevalence of these conditions.^43 48 49^ These findings highlight the importance of promoting increased adiponectin levels and improved cytokine profiles.^50^

The results also align with Sullivan *et al*,^51^ who reported that US individuals with two physical activity risk factors (sedentary for over 3 hours daily and low weekly physical activity) had twice the risk of diabetes compared with those with 0–1 physical activity risk factors. Together, this suggests low weekly physical activity and daily sedentariness may play a significant role in the onset and establishment of diabetes. Peoples’ participation in physical activity could be influenced by the built and natural environment in which they live and by personal factors such as sex, age, ability and motivation. For Sierra Leoneans to be physically active and reduce sedentary time, physical activity programmes and interventions must be proposed for urban sector-based jobs while raising awareness of the harmful effects of prolonged sitting.

Participants who consumed 2–3 sources of salt intake showed 1.8 times higher risk of obesity. After adjusting for confounders, such as location, sex and age, the current study found that 2–3 sources of salt intake were associated with a 21% higher risk of obesity. The findings agree with the broader literature that suggests high salt intake is associated with obesity.^52^,^55^ Additionally, this study adds novel insights into how behavioural risk factors manifest as physiological risk factors for CVD within Sierra Leone’s context, a setting with limited empirical evidence to date. While associations between diet, physical activity and CVD outcomes have been well-established in HICs, few nationally representative studies have assessed these relationships in low-income, postconflict settings like Sierra Leone. In the case of Sierra Leone, it is likely that the drivers behind higher salt intake and obesity could be explained by increased consumption of processed calorie-dense foods, which can lead to excessive sodium intake.^52^,^56^ Our study contributes valuable insights into the epidemiology of CVD risk factors in SSA, particularly in Sierra Leone. While causality cannot be established, our findings, along with previous literature, suggest that urban residence may be associated with increased behavioural risk factors such as processed food consumption and reduced physical activity, both of which are more common in urban settings. Sierra Leone is undergoing economic development and urbanisation, which is likely to be followed by an increase in behavioural risk factors for CVD. The findings suggest that urbanisation and associated behaviour changes, such as consumption of processed foods and low physical activity, are contributing to the rising burden of CVDs in this region. It is worth noting that dietary risk factors for CVD are highly prevalent, particularly among urban residents.^30^ Given the high prevalence of hypertension, diabetes and obesity found in this study, targeted public health strategies are urgently needed to address these risk factors. These should include prioritising urban populations in national CVD prevention efforts and improving community-level health promotion and screening. This is especially important considering the dire long-term outcomes for stroke survivors in Sierra Leone, as highlighted by Youkee *et al*,^57^ which underscores the urgent need for enhanced postdischarge care and reinforced secondary prevention strategies.

For Sierra Leone, specific interventions could include public health campaigns to raise awareness about the benefits of fruit and vegetable consumption and the risks associated with high salt intake.^58^ Policies to promote physical activity, such as creating exercise facilities and organising community fitness events, could also be beneficial.^58^ We recommend further research to explore structural and community-level enablers of healthy behaviours, and to support implementation of CVD prevention strategies tailored to local contexts. Such community-wide initiatives are crucial, as well as those focused on high-risk individuals.^58^

### Limitations of current research

This study has several limitations that must be acknowledged. First, the cross-sectional design limits our ability to infer causality, as it captures data at a single point in time and prevents establishing temporal relationships between exposure and outcome. Therefore, while associations can be identified, causation cannot be definitively determined.

Second, reliance on self-reported dietary and physical activity data introduces potential recall bias and measurement errors. Participants might not accurately remember or report their dietary intake and physical activity levels, leading to misclassification and potential bias in the findings. Although efforts were made to minimise these errors, such as using standardised questionnaires and trained data collectors, the inherent limitations of self-report data remain.

Third, the study was conducted exclusively among Sierra Leonean adults aged 40 years and above. This age restriction means that the findings may not be generalisable to younger populations or to other regions with different demographic profiles. The focus on an older population was driven by the higher prevalence of CVD risk factors in this age group, but future studies should include a broader age range to enhance generalisability.

Additionally, the study did not account for all possible confounding variables. While key sociodemographic factors were adjusted for, other potential confounders, such as genetic predispositions, environmental exposures and access to healthcare, were not included. These unmeasured confounders might have influenced the observed associations and should be considered in future research.

Finally, despite efforts to ensure accuracy, the possibility of dietary assessment measurement error by participants exists. Despite these limitations, the study provides important insights into the behavioural and physiological risk factors for CVDs in Sierra Leone and highlights areas for future research and public health interventions.

### Conclusions

In conclusion, this study reveals significant associations between dietary habits, physical activity levels and three physiological risk factors for CVD; hypertension, diabetes and obesity among adults in Bo District, Sierra Leone. The findings emphasise the critical need for targeted public health interventions aimed at promoting healthier dietary practices and increasing physical activity to mitigate the growing burden of CVDs in this region.

Addressing these modifiable risk factors through public health campaigns, policy changes and community-based interventions could significantly reduce the prevalence of CVDs and improve overall population health. Additionally, the study highlights the importance of further research to explore the complex interplay between behavioural, environmental and genetic factors in the development of CVDs.

By understanding and addressing the specific risk factors prevalent in Sierra Leone, effective strategies can be developed to combat CVDs and contribute to the global efforts to reduce the burden of NCDs. These efforts are crucial not only for improving health outcomes but also for achieving SDGs related to health and well-being in LMICs.

## Supplementary material

10.1136/bmjopen-2024-095825 online supplemental file 1

## Data Availability

Data are available on reasonable request.
