# Supplementary material for: Cross-sectional study of the association between diet and physical inactivity with obesity, diabetes and hypertension among older adults in Sierra Leone
Source: BMJ Open. 2025 Jul 1;15(7):e095825. doi: 10.1136/bmjopen-2024-095825 (PMC12215133; doi:10.1136/bmjopen-2024-095825)
Supplement: online supplemental file 1 [file bmjopen-15-7-s001.docx]

**Supplemental Material**

**Prevalence of CVD Risk Factors by individual Behavioural factors**

Participants who consumed fewer than five servings of fruits and vegetables daily exhibited higher prevalence of hypertension (85.8% vs. 14.2%), diabetes (89% vs. 10.4%), and overweight/obesity (84.7% vs. 15.3%) compared with those who met the recommended intake of five servings per day (Table 1).

In contrast, participants with higher salt intake, defined as consuming salt from two or three sources, demonstrated a lower prevalence of hypertension (46.1% vs. 53.9%) and overweight/obesity (47.1% vs. 52.9%) but a higher prevalence of diabetes (51.1% vs. 42.9%) compared with those with lower salt intake.

Physical activity also had a significant association with the prevalence of physiological CVD risk factors. Individuals with at least one physical activity risk factor (e.g., inadequate moderate and vigorous physical activity (MVPA) or high levels of physical inactivity) showed higher prevalence rates of hypertension (42.0% vs. 40.5%), diabetes (40.8% vs. 38.9%), and overweight/obesity (44.4% vs. 41.8%) compared with those with no physical activity risk factors (Table 1).

**Table 1: Descriptive Analysis of Behavioural Risk Factors and CVD Physiological Risk Factors**

| **Risk factors** | **No HTN n(%)** | **HTN n(%)** | **No DM n(%)** | **DM n(%)** | **Normal weight n(%)** | **Overweight/Obese n(%)** |
| --- | --- | --- | --- | --- | --- | --- |
| **Fruit and Vegetables** |  |  |  |  |  |  |
| >5FVeg | 6762 (19.1) | 4409 (14.2) | 10494 (16.6) | 165 (10.4) | 7590 (16.7) | 2786 (15.3) |
| <5FVeg | 28557 (80.9) | 26708 (85.8) | 52615 (83.4) | 1423 (89.6) | 37828 (83.3) | 15469 (84.7) |
| **Salt Intake** |  |  |  |  |  |  |
| <1 source of salt intake | 28030 (56.3) | 25264 (53.9) | 51638 (56.1) | 1096 (42.9) | 37530 (55.8) | 13742 (52.9) |
| 2 or 3 sources of salt intake | 21762 (43.7) | 21598 (46.1) | 40401 (53.4) | 1461 (57.1) | 29705 (44.2) | 12256 (47.1) |
| **Physical Activity Risk Factors** |  |  |  |  |  |  |
| 0 Physical Activity risk factors | 25560 (51.3) | 19010 (40.5) | 42907 (46.6) | 1011 (38.9) | 32666 (48.6) | 10866 (41.8) |
| 1 Physical Activity risk factors | 20081 (40.3) | 19681 (42.0) | 37562 (40.8) | 1060 (40.8) | 27238 (40.5) | 11555 (44.4) |
| 2 Physical Activity risk factors | 4151 (8.3) | 8213 (17.5) | 11571 (12.6) | 528 (20.3) | 7330 (10.9) | 3578 (13.8) |

Sociodemographic characteristics were associated with the prevalence of physiological CVD risk factors. Rural residents had a higher prevalence of diabetes and overweight/obesity, but a lower prevalence of hypertension compared with urban residents (Table 5). This variation may reflect differences in behaviour and access to healthcare services between rural and urban areas.

Sex differences were also apparent, with males exhibiting lower prevalence rates of hypertension (46.2% vs. 53.8%), diabetes (45.9% vs. 54.1%), and overweight/obesity (33.3% vs. 66.7%) compared with females. Age-wise, individuals aged 40-49 years had higher prevalence rates of hypertension (32.4%), diabetes (31.1%), and overweight/obesity (47.9%) compared to older age groups (Table 2). This suggests that middle-aged adults in this cohort may be at greater risk and highlights the need for targeted interventions.

**Table 2: Descriptive Analysis of Sociodemographic Characteristics by Hypertension, Diabetes, and Weight Status**

|  | **No HTN n(%)** | **HTN n(%)** | **No DM n(%)** | **DM n(%)** | **Normal weight n(%)** | **Overweight/Obese n(%)** |
| --- | --- | --- | --- | --- | --- | --- |
| **Location** |  |  |  |  |  |  |
| Rural | 33526 (67.3) | 27045 (57.7) | 57713 (62.7) | 1214 (46.7) | 45916 (68.3) | 12881 (49.5) |
| Urban | 16266 (32.7) | 19858 (42.3) | 34327 (32.3) | 1384 (53.3) | 21319 (31.7) | 13117 (50.5) |
| **Sex** |  |  |  |  |  |  |
| Female | 22432 (45.1) | 25242 (53.8) | 45346 (49.3) | 1406 (54.1) | 28470 (42.3) | 17336 (66.7) |
| Male | 27361 (54.9) | 21661 (46.2) | 46693 (50.7) | 1192 (45.9) | 38765 (57.7) | 8663 (33.3) |
| **Age Group** |  |  |  |  |  |  |
| 40-49 | 27799 (55.8) | 15185 (32.4) | 41187 (44.7) | 807 (31.1) | 29368 (43.7) | 12461 (47.9) |
| 50-59 | 11711 (23.5) | 12578 (26.8) | 23237 (25.2) | 578 (22.3) | 17084 (25.4) | 6564 (25.2) |
| 60-69 | 5583 (11.2) | 8761 (18.7) | 13413 (14.6) | 671 (25.8) | 9912 (14.7) | 4009 (15.4) |
| 70-79 | 2802 (5.6) | 6112 (13.0) | 8349 (9.1) | 425 (16.4) | 6495 (9.7) | 2000 (7.7) |
| 80+ | 1899 (3.8) | 4268 (9.1) | 5854 (6.4) | 116 (4.5) | 4377 (6.5) | 965 (3.7) |

**Association Between Behavioural Risk Factors, Sociodemographic Characteristics, and Physiological Risk Factors for CVD**

The results from the unadjusted regression analysis reveal that individuals consuming fewer than five servings of fruits and vegetables daily had significantly higher odds of hypertension (OR=1.62, 95% CI [1.56-1.69], p<0.001), diabetes (OR=1.82, 95% CI [1.54-2.15], p<0.001), and overweight/obesity (OR=1.17, 95% CI [1.12-1.23], p<0.001) compared with those meeting the recommended intake (Table 3).

In terms of salt intake, participants consuming salt from two or three sources had higher odds of diabetes (OR=1.36, 95% CI [1.23-1.51], p<0.001) and overweight/obesity (OR=1.18, 95% CI [1.14-1.22], p<0.001), but lower odds of hypertension (OR=0.94, 95% CI [0.91-0.97], p<0.001) compared with those with lower salt intake. For physical activity, individuals with two physical activity risk factors had significantly higher odds of hypertension (OR=2.40, 95% CI [2.27-2.52], p<0.001), diabetes (OR=2.31, 95% CI [2.01-2.65], p<0.001), and overweight/obesity (OR=1.26, 95% CI [1.19-1.34], p<0.001) compared with those with no physical activity risk factors (Table 3).

**Table 3: Unadjusted Logistic Regression Models for Behavioural Risk Factors and Physiological Conditions**

| **Risk Factors** | **Hypertension**  OR  (95% CI) | **Diabetes**  OR  (95% CI) | **Overweight/Obesity**  OR  (95% CI) |
| --- | --- | --- | --- |
| **Fruit and Vegetables** |  |  |  |
| >5FVeg | Referent | Referent | Referent |
| <5FVeg | 1.62*** (1.56-1.69) | 1.82*** (1.54-2.15) | 1.17*** (1.12-1.23) |
| **Salt Intake** |  |  |  |
| <1 source of salt intake | Referent | Referent | Referent |
| 2 or 3 sources of salt intake | 0.94*** (0.91-0.97) | 1.36*** (1.23-1.51) | 1.18*** (1.14-1.22) |
| **Physical Activity Risk factors** |  |  |  |
| 0 Physical Activity risk factors | Referent | Referent | Referent |
| 1 Physical Activity risk factors | 1.40*** (1.35-1.45) | 1.20*** (1.07-1.35) | 1.22*** (1.17-1.26) |
| 2 Physical Activity risk factors | 2.40*** (2.27-2.52) | 2.31*** (2.01-2.65) | 1.26*** (1.19-1.34) |
