# Supplementary material for: Cross-sectional study of the association between diet and physical inactivity with obesity, diabetes and hypertension among older adults in Sierra Leone
Source: BMJ Open. 2025 Jul 1;15(7):e095825. doi: 10.1136/bmjopen-2024-095825 (PMC12215133; doi:10.1136/bmjopen-2024-095825)
Supplement: Supplementary file 2 [file bmjopen-2024-095825.reviewer_comments.pdf]

PEER REVIEW HISTORY

BMJ Open publishes all reviews undertaken for accepted manuscripts. Reviewers are asked to complete a checklist review form and are provided with free text boxes to elaborate on their assessment. These free text comments are reproduced below.

ARTICLE DETAILS

Title (Provisional)

A cross-sectional study of the association between diet and physical inactivity with obesity, diabetes, and hypertension among older adults in Sierra Leone.

Authors

Bockarie, Tahir; Shanker, Ankit; Jalloh, Mohamed B.; Kamara, Alhaji M.; Odland, Maria Lisa; Wurie, Haja; Ansumana, Rashid; Lamin, Joseph; Witham, Miles; Oyebode, Oyinlola; Davies, Justine

---

VERSION 1 - REVIEW

**Reviewer**	**1**
**Name**	**Fock, Kwong Ming**
**Affiliation**	**Changi Gen Hosp**
**Date**	**27-Dec-2024**
**COI**	**None**

This manuscript is a study on the relationship between diet, physcical inactivity and CVD risk factors in Sierra Leone. Study objectives were clearly stated .Population involved in the survey included urban and rural residents. Sampling was carefully chosen . Results were within expectations.Minor point: overweight is defined as BMI >25 .Obeisty is defined as BMI. >30.This study does help to shape public health policy in Sieera Leone.

**Reviewer**	**2**
**Name**	**Goodwin, Ashley**
**Affiliation**	**Northwell Health Feinstein Institutes for Medical Research, Institute for Health System Science**
**Date**	**08-Apr-2025**
**COI**	**None**

Thank you for the opportunity to review the manuscript 'The association of diet and physical inactivity with obesity, diabetes, and hypertension 2 among older adults living in Sierra

Leone'. In this study, Bockarie et al sought to assess the association between behavioral risk factors, namely diet, physical activity, and salt intake, and physiological risk factors for cardiovascular disease, namely hypertension, diabetes, and obesity, among adults aged 40 and above in Bo District, Sierra Leone. The authors utilized a community-based cross-sectional study design conducted in Bo District, Sierra Leone, to administer a household survey based on the validated WHO STEPs questionnaire. The primary outcome was presence of hypertension, diabetes, and obesity (CVD risk factors). Results revealed that at least one physiological risk-factor for CVD was present in 43.5% of participants, and that presence of each risk factor was associated with at least one behavioral risk factor. The authors conclude that the prevalence and these associations highlight a public health issue that warrants targeted intervention efforts to promote healthier lifestyles to reduce CVD prevalence among the population in Sierra Leone.

Overall, the manuscript would benefit from revisions for copyediting (e.g., various grammatical and formatting errors), and to clarify key concepts/study objective, remove redundancies, and elaborate on the outcomes. The importance of the work is clear, as less is known about the behavioral risk factors for CVDs in this area alongside a rise in the prevalence of CVDs. In general, the objective of the study needs to be clearer, results need to be interpreted more substantively, and the discussion needs to offer more than a re-statement of the results, in the context of the extant literature. It is not clear what this study is adding or how it has extended the findings. Based on the data reported, there appears to be a robust dataset to interpret and draw conclusions from. The authors are encouraged to elaborate and interpret in order to bring the manuscript up to the level that warrants publication. Below are comments notated by manuscript section and line number.

Abstract:

Line 46: Can you include sample characteristics in parentheses, such as %male or female, age range, etc?

Line 50: if diet, physical activity and salt intake are the only three factors, 'such as' is not necessary; it also is slightly confusing to the reader who is left wondering what other factors may have been explored (e.g., smoking)

Line 53: Primary outcome is vague. More detail regarding the primary outcome would be helpful (i.e., was it a simple 'yes/no' on a self-report survey?). For example, could you say "self-reported presence of hypertension, diabetes, and obesity" or "confirmed presence of hypertension, diabetes, and obesity"? Also, consider revising 'and' to 'or' or 'and/or', as it is written it appears that all three would be present. Lastly, is the primary outcome actually the presence of these risk factors, or the association of the risk factors with the behavioral risk factors? This needs to be clarified

Line 55: This is the first mention of 'CVD'; this should be spelled out for the first use, and also the context to CVD should be included in the objective so that it does not seem to come out of nowhere in the results.

Line 62: I would expect the results for obesity to be reported similar to hypertension and diabetes (i.e. their association with behavioral risk factors). Was there no association with behavioral risk factors? What about 'likelihood' for other demographic factors for hypertension and diabetes? Consider reporting results consistently for clarity.

Line 64: The conclusion is vague. Can the authors comment more substantively on their results?

The strengths and limitations section does a nice job of summarizing the work.

Introduction

Line 80: What is Sustainable Development Goal 3?

Line 82: This sentence is unclear, perhaps due to grammatical errors

Lines 84-86: Inclusion of systematic reviews or other analyses from the literature would strengthen this statement. Why is it important to understand the relationship between behavioral and physiological CVD risk factors? If this is the crux of the study, it is important to support this concept empirically.

Line 96: duplicate citations

Lines 96-98: What is the citation for this?

Lines 98-99: This is redundant with lines 94-96; this paragraph would benefit from revision for clarity

Lines 106-108: What studies? This statement requires a citation to the works referenced. What were the conclusions of these studies? How will this study extend the findings?

Line 112: What does an 'epidemiological transition' mean in the context of this study?

Line 114: extraneous period

Methods:

A consort diagram would help the reader understand the sample size, as various numbers are reported. For example, in the abstract a sample of 1,978 was reported, but in the methods section 2,071 individuals are reported to have been recruited (line 137). Then, in a following paragraph (starting on line 145), if you add up the numbers detailed in the sampling strategy, the total is 1,351 participants (7 urban communities with 100 recruited in each, plus 7 rural communities with 93 participants each). It is not clear how the sample reached 2,071 based on this accounting. If the final sample analyzed was 1,978, the authors should provide an account of why the others recruited were not included (e.g., missing data, etc).

Line 132: 'with a specific focus on diabetes prevalence' -- why was this the case? I would expect this to have been covered in the introduction

Line 149: why is 'Seven' capitalized?

Line 156: incomplete sentence

Lines 156-157: Again, a consort diagram is necessary to understand the sample

Line 200: Why is Sex capitalized?

Line 228: 'less than 3 hours and 3 or more hours' -- How was this cut point determined?

Line 254: What level of missing data?

Line 261: 'lifestyle factors and clinical outcomes' -- please use consistent terms throughout the manuscript. Up to this point, these were called behavioral risk factors and CVD risk factors.

Line 269: It is not clear what the weighted population is

Line 280: Is this considered Figure 1? Also, why not use the unweighted sample for the y-axis? Or have Figure 1a and 1b, one with weighted and one with non-weighted. It also appears that zero males and zero rural individuals had 3 risk factors, but this is not the case in Table 2 (suspect this is due to using the weighted sample and makes the case for the previous suggestion)

Table 1: Revising formatting to bold sub-headings would improve readability (as in Table 4)

Table 2: It's clear how the sample is stratified by number of CVD risk factors, but it would be helpful to add which factors. For example, individuals who had 2 risk factors could be further broken down to hypertension and diabetes, diabetes and obesity, and hypertension and obesity.

Table 3: It is not clear why diabetes is left out of this table

Line 295: 'dose-response' is not the right term to use here

Discussion:

Line 336: 'physiological risk factors for hypertension, diabetes, and obesity' - wouldn't it be physiological risk factors for CVD (as it is stated throughout the paper)? Hypertension, diabetes, and obesity are the risk factors for CVD

Line 340: Cite the literature being referenced, and place the results into context with extant literature

Lines 348-352: Could the authors expand on these results, and offer (or hypothesize) a more substantive reason for the surprising result, based on extant literature?

Lines 353-374: What is this study adding to the existing evidence? How do these results extend current findings? Much of the discussion is either re-stating what is in the results section without interpretation, or stating that findings are in line with what has already been established. Was the purpose of the study to determine (confirm?) if the population in Sierra Leone followed similar associations between behavioral and physiological risk factors, to inform public policy? If so, this needs to be much more clear. As it is written, it is not clear

what this study is adding. The discussion would benefit from the addition of interpretation of the results.

Line 376: 'suggest that urbanization' - how do we know that it is because of urbanization? Could it not be other factors? This appears to be conjecture, as I am not convinced the evidence can be tied to urbanization directly, without supporting evidence that suggests otherwise.

Line 385; Lines 420-422: The mention of the environment and genetic factors does not make sense in the context the findings. In general, the authors need to make a much more solid connection between the results and the implications; a lot of the statements appear as opinions.

Line 408: This is redundant with lines 394-395.

VERSION 1 - AUTHOR RESPONSE

Reviewer 1

Comment: This manuscript is a study on the relationship between diet, physical inactivity and CVD risk factors in Sierra Leone. Study objectives were clearly stated. Population involved in the survey included urban and rural residents. Sampling was carefully chosen. Results were within expectations. Minor point: overweight is defined as BMI >25. Obesity is defined as BMI >30. This study does help to shape public health policy in Sierra Leone.

Response: We thank the reviewer for the positive feedback and acknowledgement of the study's public health relevance. We have corrected the minor issue regarding the definition of overweight and obesity. The manuscript now clearly defines overweight as BMI ≥25 and obesity as BMI ≥30, in line with WHO standards. (See Methods section)

Reviewer 2

General Comment: The manuscript explores an important and timely topic. However, several areas require improvement, including clarification of objectives, elaboration on outcomes, reduction of redundancies, stronger interpretation in the discussion, and consistency in formatting.

Response: We thank the reviewer for their detailed and constructive comments. Below we respond to each specific point and have revised the manuscript accordingly.

Abstract:

- Line 51-52: Can you include sample characteristics in parentheses, such as %male or female, age range, etc?
  Comment: Sample characteristics have been added (age range and gender distribution) to improve clarity. The revised abstract includes age range (40+) and sex distribution

- Line 56: if diet, physical activity and salt intake are the only three factors, 'such as' is not necessary; it also is slightly confusing to the reader who is left wondering what other factors may have been explored (e.g., smoking)
  Comment: The phrase "such as" has been removed to prevent confusion, as only diet, physical activity, and salt intake were assessed.

- Line 58: Primary outcome is vague. More detail regarding the primary outcome would be helpful (i.e., was it a simple 'yes/no' on a self-report survey?). For example, could you say "self-reported presence of hypertension, diabetes, and obesity" or "confirmed presence of hypertension, diabetes, and obesity"? Also, consider revising 'and' to 'or' or 'and/or', as it is written it appears that all three would be present. Lastly, is the primary outcome actually the presence of these risk factors, or the association of the risk factors with the behavioral risk factors? This needs to be clarified
  Comment: The primary outcome has been clarified as the "presence of hypertension, diabetes, and/or obesity," and it is now stated whether these were self-reported or clinically assessed.

- Line 65: This is the first mention of 'CVD'; this should be spelled out for the first use, and also the context to CVD should be included in the objective so that it does not seem to come out of nowhere in the results.
  Comment: The term "CVD" is now spelled out in full on first use and better contextualized within the study objective.

- Line 72: I would expect the results for obesity to be reported similar to hypertension and diabetes (i.e. their association with behavioral risk factors). Was there no association with behavioral risk factors? What about 'likelihood' for other demographic factors for hypertension and diabetes? Consider reporting results consistently for clarity.
  Comment: Obesity-related associations have been added to the abstract to align with how hypertension and diabetes results were presented.

- Line 64: The conclusion is vague. Can the authors comment more substantively on their results?
  Comment: The conclusion in the abstract has been strengthened to more substantively reflect the implications of the findings.

Introduction:

- Line 109: What is Sustainable Development Goal 3?
  Comment: A brief explanation of Sustainable Development Goal 3 has been added.
- Line 111: This sentence is unclear, perhaps due to grammatical errors
  Comment: The sentence has been rewritten for clarity and grammar.
- Lines 116-120: Inclusion of systematic reviews or other analyses from the literature would strengthen this statement. Why is it important to understand the relationship between behavioural and physiological CVD risk factors? If this is the crux of the study, it is important to support this concept empirically.

Comment: A recent systematic review was cited to empirically support the relevance of understanding behavioural and physiological risk factor relationships (Reference: Roth GA, Mensah GA, Johnson CO, et al. Global Burden of Cardiovascular Diseases and Risk Factors, 1990–2019: Update from the GBD 2019 Study. *Journal of the American College of Cardiology* 2020;76(25):2982-3021. doi: https://doi.org/10.1016/j.jacc.2020.11.010).

- Line 126 & 128: Duplicate citations
  Comment: Duplicate and unclear citations were removed or corrected.
  Lines 131–135: What studies? This statement requires a citation to the works referenced. What were the conclusions of these studies? How will this study extend the findings?
  Comment: Specific studies are now cited and summarized to show how this study builds upon previous findings.
- Line 112: What does an 'epidemiological transition' mean in the context of this study?
  Comment: The concept of "epidemiological transition" has been removed from current draft.
- Line 114: Extraneous period
  Comment: The extraneous period has been deleted.

Methods:

- Comment: A consort diagram would help the reader understand the sample size, as various numbers are reported. For example, in the abstract a sample of 1,978 was reported, but in the methods section 2,071 individuals are reported to have been recruited (line 137). Then, in a following paragraph (starting on line 145), if you add up the numbers detailed in the sampling strategy, the total is 1,351 participants (7 urban communities with 100 recruited in each, plus 7 rural communities with 93 participants each). It is not clear how the sample reached 2,071 based on this accounting. If the final sample analyzed was 1,978, the authors should provide an account of why the others recruited were not included (e.g., missing data, etc).
  Response: On line 183, a participant flow description has been added in the Methods, and a visual flow diagram (Figure 1) is included in the revised submission to clearly outline recruitment, exclusions, and final sample size.
- Line 163: 'with a specific focus on diabetes prevalence' -- why was this the case? I would expect this to have been covered in the introduction
  Response: We have now clarified in Methods sections that diabetes was anticipated to be the least prevalent of the three physiological risk factors, and therefore drove the sample size calculation to ensure adequate power to detect its prevalence with ±1% precision.
- Line 197: why is 'Seven' capitalized?
  Response: These capitalization inconsistencies have been corrected throughout the manuscript.
- Line 204: incomplete sentence
  Response: All identified incomplete or unclear sentences have been revised for clarity and grammatical accuracy.

- Lines 204-206: Again, a consort diagram is necessary to understand the sample
  Response: Sample diagram created and now feature under ‘Figure 1’.
- Line 247: Why is Sex capitalized?
- Response: These capitalization inconsistencies have been corrected throughout the manuscript.
- Line 275: 'less than 3 hours and 3 or more hours' -- How was this cut point determined?
- Response: The manuscript has clarified that the 3-hour threshold was selected based on previous studies (references 28 and 29) which have shown that sedentary behaviour exceeding 3 hours per day is associated with elevated cardiometabolic risk. This cut-off is now explicitly justified in the revised text.
- Line 310-313: It is not clear what the weighted population is
  Response: The manuscript now includes a detailed explanation of the statistical weighting approach used to ensure representativeness, with weights based on the 2015 census.
- Line 323: What level of missing data?
  Response: The manuscript now clearly states that 93 participants (4.5%) were excluded due to missing data, resulting in a final sample of 1,978.
- Line 334: Is this considered Figure 1? Also, why not use the unweighted sample for the y-axis? Or have Figure 1a and 1b, one with weighted and one with non-weighted. It also appears that zero males and zero rural individuals had 3 risk factors, but this is not the case in Table 2 (suspect this is due to using the weighted sample and makes the case for the previous suggestion)
  Response: In the revised manuscript, Figure 1 is now the participant flow diagram, and the bar chart has been updated as Figure 2, titled “Distribution of CVD Physiological Risk Factors by Demographics.” Figure 2 presents weighted data to reflect population-level estimates. To clarify the appearance of zero values for subgroups like males and rural residents with 3 risk factors: these are due to rounding in the weighted data. Actual cases are present, as shown in Table 2, which reports unweighted counts. This clarification has been added to both the figure legend and methods section.
- Table 1: Revising formatting to bold sub-headings would improve readability (as in Table 4)
  Response: We have revised the formatting of Table 1to bold the sub-headings, consistent with the style used in Table 4, to improve readability and visual alignment across tables.
- Table 2: It's clear how the sample is stratified by number of CVD risk factors, but it would be helpful to add which factors. For example, individuals who had 2 risk factors could be further broken down to hypertension and diabetes, diabetes and obesity, and hypertension and obesity.
- Response: Thank you for this helpful suggestion. While Table 2 summarizes the number of co-occurring CVD physiological risk factors by demographic groups, we agree that providing details on specific combinations (e.g., hypertension + diabetes) would offer additional insights. To preserve the clarity of the main table, we have retained its current format but now note this limitation in the revised Results and Discussion sections.
- Table 3: It is not clear why diabetes is left out of this table

Response: Diabetes was excluded from Table 3 because no statistically significant associations were observed between the number of behavioural risk factors and the presence of diabetes across all categories. This is now explicitly clarified in both the note beneath Table 3 and in the Results section to improve transparency and avoid confusion.

- Line 347: 'dose-response' is not the right term to use here
  Response: The term 'dose-reponse' has been removed from the manuscript and replaced with more appropriate wording (dose-dependent) to accurately reflect the observed trend without implying causality.

Discussion:

- Line 388: 'physiological risk factors for hypertension, diabetes, and obesity' - wouldn't it be physiological risk factors for CVD (as it is stated throughout the paper)? Hypertension, diabetes, and obesity are the risk factors for CVD
  Response: We agree with the reviewer's observation and have revised the phrase to accurately reflect that *hypertension, diabetes, and obesity are physiological risk factors for cardiovascular disease (CVD)*, not for each other. The wording has been corrected throughout the manuscript to maintain consistency.
- Line 397: Cite the literature being referenced, and place the results into context with extant literature
  Response: We have revised the sentence to include relevant citations and positioned the findings in the context of existing literature.
- Lines 407-422: Could the authors expand on these results, and offer (or hypothesize) a more substantive reason for the surprising result, based on extant literature?
  Response: We have expanded the discussion on Lines 407–422 to offer a more substantive hypothesis explaining the counterintuitive finding related to salt intake and hypertension. We now draw on existing literature to suggest possible explanations such as reverse causality, reporting bias, co-occurring dietary behaviours, and individual variability in salt sensitivity. Relevant citations have been included to support these points and place the finding in a broader epidemiological context.
- Lines 435-455: What is this study adding to the existing evidence? How do these results extend current findings? Much of the discussion is either re-stating what is in the results section without interpretation, or stating that findings are in line with what has already been established. Was the purpose of the study to determine (confirm?) if the population in Sierra Leone followed similar associations between behavioural and physiological risk factors, to inform public policy? If so, this needs to be much more clear. As it is written, it is not clear what this study is adding. The discussion would benefit from the addition of interpretation of the results.
  Response: In response, we have revised the Discussion section to explicitly state the novel contribution of our study; namely, that it provides one of the first nationally representative assessments of behavioural and physiological CVD risk factor associations in Sierra Leone. We now clarify that the aim was to examine whether these associations, well documented globally, are also

observed in this setting, and how they can inform public health strategies. We have also added interpretation to contextualize the findings in terms of local urbanization, food systems, and behaviour change potential, and we have removed redundant repetition of results.

- Line 446: 'suggest that urbanization' - how do we know that it is because of urbanization? Could it not be other factors? This appears to be conjecture, as I am not convinced the evidence can be tied to urbanization directly, without supporting evidence that suggests otherwise.
  Response: We agree that our previous wording could imply causation without sufficient support. We have revised the text to clarify that we are not making a direct causal claim about urbanization but instead highlighting an observed association between urban residence and CVD risk factors, supported by both our data and broader literature.
- Lines 466-467: The mention of the environment and genetic factors does not make sense in the context the findings. In general, the authors need to make a much more solid connection between the results and the implications; a lot of the statements appear as opinions.
  Response: We have revised the discussion section to remove unsupported references to genetic and environmental factors. Instead, we now emphasize structural and contextual determinants that are more directly linked to the study's findings. We also clarified the connection between our results and their implications for public health interventions in Sierra Leone.
- Line 465: This is redundant with lines 465-485.
  Response: We have removed the redundant sentence referencing behavioural, environmental, and genetic factors from the *Discussion* section. In its place, we now emphasize the need for research into structural and community-level enablers of healthy behaviours, with a focus on implementation of locally tailored CVD prevention strategies.

We hope these comprehensive revisions meet the expectations of the reviewer and strengthen the manuscript.
